# Supplementary material for: Cryo-EM unveils kinesin KIF1A’s processivity mechanism and the impact of its pathogenic variant P305L
Source: Nat Commun. 2024 Jul 2;15:5530. doi: 10.1038/s41467-024-48720-4 (PMC11219953; doi:10.1038/s41467-024-48720-4)
Supplement: Supplementary file 1 — Supplementary Information [file 41467_2024_48720_MOESM1_ESM.pdf]

Cryo-EM Unveils Kinesin KIF1A's Processivity Mechanism and the Impact of its Pathogenic Variant P305L

Matthieu P.M.H. Benoit[1,*,#], Lu Rao[1,*], Ana B. Asenjo[1], Arne Gennerich[1,#], and Hernando Sosa[1,#]

Supplementary Figures and Tables

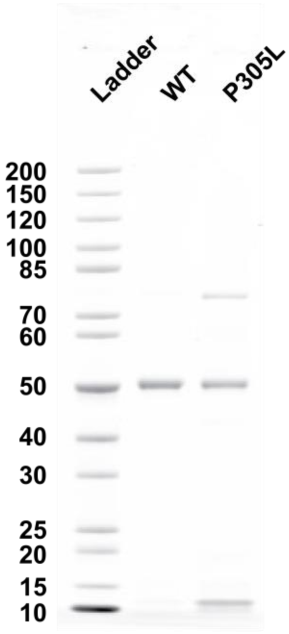

Supplementary Fig 1. Polyacrylamide gel of KIF1A constructs. Coomassie blue-stained gel of KIF1A (KIF1A(aa1-393)-LZ-strepII) (WT) and KIF1A^{P305L}(KIF1A(aa1-393, P305L)-LZ-strepII) (P305L); Ladder: molecular weight standards. The molecular weight of the ladder is indicated on the left in kDa.

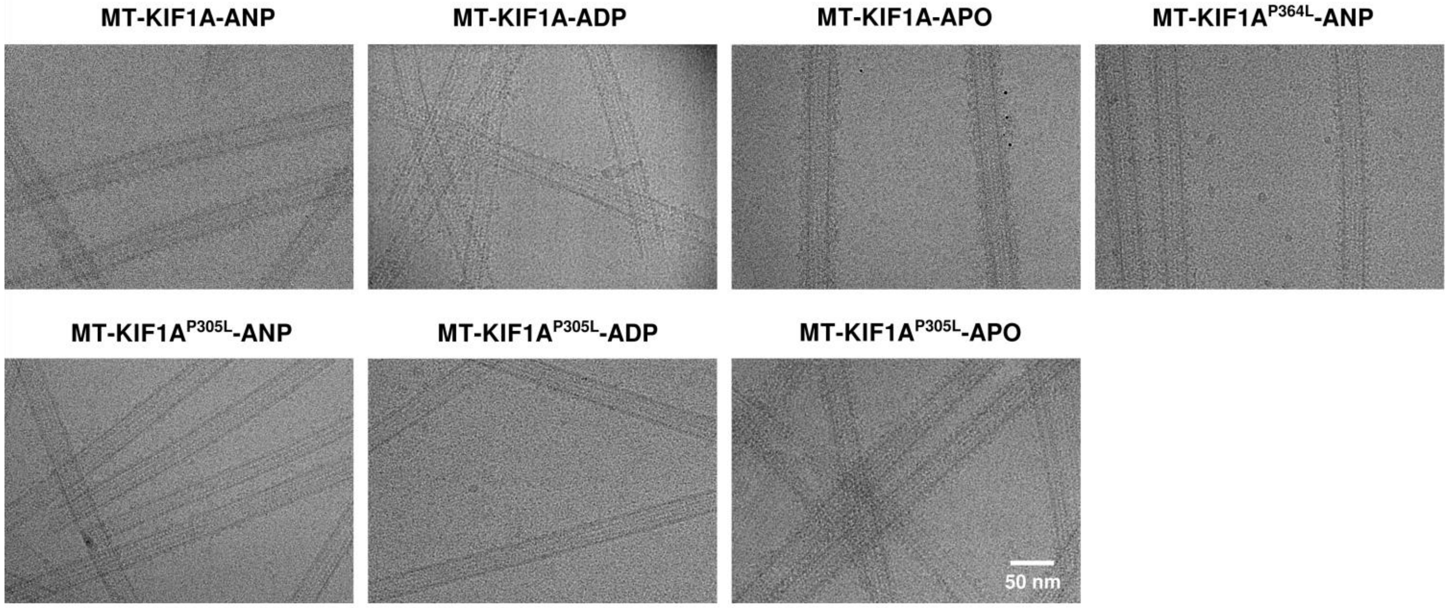

Supplementary Fig 2. Representative micrographs. Each panel shows a sample micrograph (average of aligned movie frames) from each of the indicated datasets.

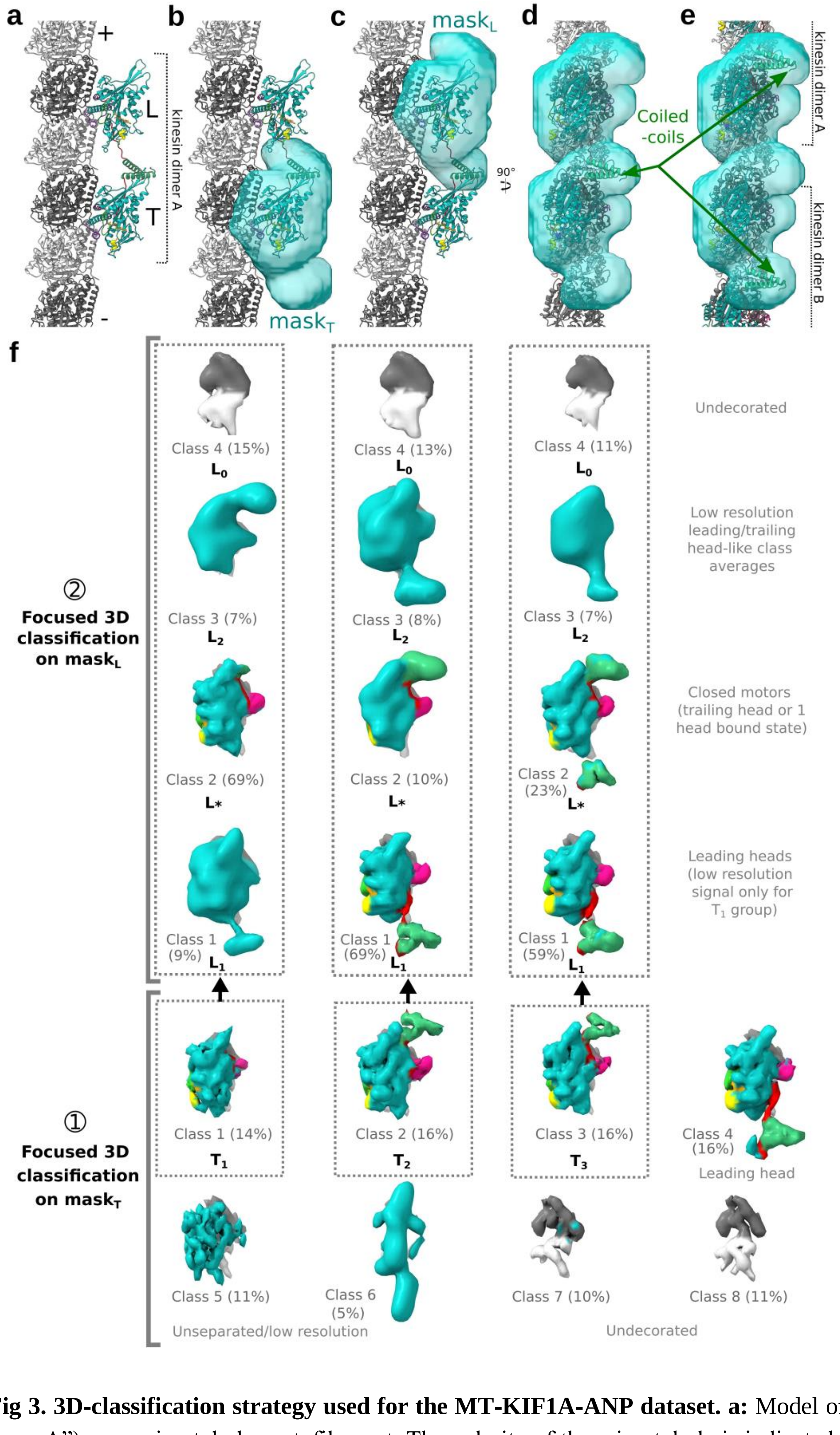

Supplementary Fig 3. 3D-classification strategy used for the MT-KIF1A-ANP dataset. a: Model of a KIF1A kinesin dimer ("kinesin dimer A") on a microtubule protofilament. The polarity of the microtubule is indicated. The leading head of the kinesin dimer is indicated by the letter L while the trailing head is indicated by the letter T. Coiled-coil is colored green and other colors are as in Fig.1. **b-c:** Semi-transparent surface view of the two masks mask$_T$ (**b**) and mask$_L$ (**c**) used successively during the 3D classification. These two 30 Å-resolution masks differ only by their position: mask$_T$ covers the trailing position of the kinesin dimer A (site T, **b**), while mask$_L$ covers the leading position (site L, **c**) at the next kinesin-

binding site toward the (+) end of the microtubule protofilament. **d:** 90 degree rotated view of the kinesin dimer model of panel (**a**) with both $mask_T$ and $mask_L$ displayed. **e:** Same view as (**d**) with 2 consecutive two-heads-bound kinesin dimers A and B attached to the microtubule protofilament but with a registration off by 8 nm (length of tubulin dimer) compared to the kinesin dimer A shown in (**a-e**). Note that both masks cover the area occupied by the coiled-coil densities for the two types of registrations displayed in (**e**) and (**d**). **f:** Scheme representing the classification strategy used for the MT-KIF1A-ANP dataset using the $mask_T$ and $mask_L$ illustrated in (**a-e**). All class averages shown in the panel correspond to the 3.5Å/pixel class averages produced by the 3D classifications, maintaining the same viewing orientation as panels (**d-e**). In the initial step (step ①), a 3D classification in 8 classes and focusing on $mask_T$ was performed. This led to the 8 class averages displayed on the lower part of the figure. The classes 1-3 correspond to kinesin motors in closed conformationss with the neck-linker docked, obtained in approximately equal proportions (refer to Supplementary Fig. 4 for a full-resolution side view of these three classes). These classes have been designated as T_1, T_2 and T_3. Class 4 corresponds to a leading head configuration, featuring an undocked neck-linker pointing backwards and connected to a coiled-coil structure. Class 5 contains a kinesin-like density but its specific state could not be confidentially assigned. It likely represents particles for which the state has not been effectively separated, unlike those classes 1 to 4. Class 6 corresponds to a low-resolution class for which a state could not be assigned. In contrast, classes 7 and 8 are devoid of any distinct decoration. Due to the symmetry expansion employed, the leading heads observed in the two-heads-bound configuration, as exemplified by class 4, are also expected to be present on site L. Given this, and considering that only the classes T_1, T_2 and T_3 could correspond to two-heads-bound configuration kinesin dimers with their leading head on site L, these three classes were further classified on site L. In the second classification step (step ②), with a focus on $mask_L$, the particles from each of the T_1, T_2 and T_3 classes were further divided into four subclasses. The corresponding class averages are presented in the figure. These class averages can be grouped into four distinct categories, as indicated in the figure, with some differences in relative frequencies. Each of T_1, T_2 and T_3 has a major class at position L, containing respectively 69%, 69% and 59% of their particles. This major class, denoted as 'L_*', represents a motor in a closed conformation (i.e., belonging to another dimer) for T_1 (specifically, class-2 in T_1 classification at site L with $mask_L$). It is worth noting that unless the kinesin coiled-coil were to unfold, the motor observed on the site L must correspond to another kinesin molecule. This indicates that the kinesin motor on site T, as seen in both class T_1 and sub-class L_* (abbreviated T_1L_*), exists in a one-head-bound configuration. In contrast to T_1, for T_2 and T_3, the major class observed on site L represents a connected leading head that is part of a two-heads-bound kinesin configuration. These classes, characterized by connected leading heads, were designated as 'L_1'. In the T_1 classification on site L, only a weak and low-resolution class exhibited similarity to a leading head (Class 1, named L_1 ; see also Supplementary Fig. 4 for a full-size reconstruction of this class), so T_1L_1 does not appear to be a pure two-heads-bound configuration class, unlike T_2L_1 and T_3L_1. Each of the classifications on site L for T_2 and T_3 also includes a class (class 2) corresponding to motors in a closed conformation with a coiled-coil density towards the (+) end of the motor (see Supplementary Fig. 4). Similar to the T_1 classification, these classes were named L_* and they indicate the presence of one-head-bound configurations on the T site. In addition, each classification on site L from T_1, T_2 and T_3, yielded other low-resolution classes resembling either leading or trailing-head-like classes (classes 3, named L_2), as well as empty sites (classes 4, named L_0) without kinesin densities. The presence of these empty sites on site L indicates that the corresponding motors on site T (classes T_1L_0, T_2L_0 and T_3L_0) are in a one-head-bound configuration.

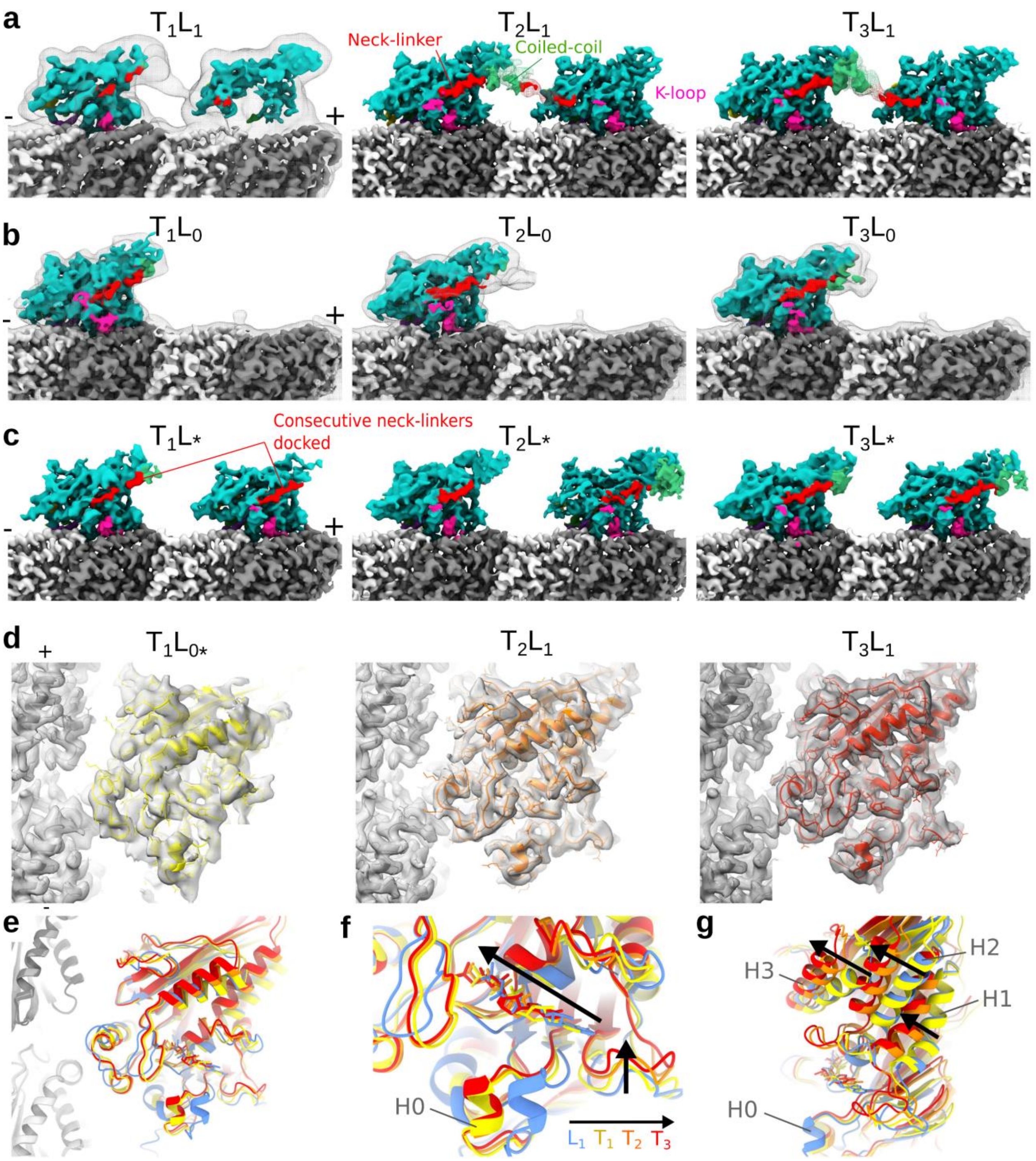

Supplementary Fig 4. Multiple binding modes and conformational states of KIF1A-ANP. a-c: Isosurface representations of the cryo-EM maps from the KIF1A-ANP dataset for the classes T_1L_1, T_2L_1 and T_3L_1 (**a**), T_1L_0, T_2L_0 and T_3L_0 (**b**), T_1L_*, T_2L_* and T_3L_*. **c**: The color scheme is the same as the one used in Fig. 1. The polarities of the microtubule protofilaments are indicated. To visualize the mobile K-loop region better, the signal around it was low-passed filtered and displayed at a lower contour level compared to the rest of the map. It's important to highlight that in (**a**), the motor domain at site L exhibits a weaker signal compared to the one at site T. Only through low-pass filtering of this class average, as illustrated by the gray mesh, was it possible to detect a weak signal of connectivity between the two heads. Therefore, the small T_1L_1 class, accounting for 9% of the site L classification within the T_1 class, appears to be a heterogeneous class that cannot be confidently assigned as being a two-heads-bound configuration. Since the other T_1 classes are in a one-head-bound configuration, the vast majority of the T1 motors are therefore in a one-head-bound configuration, unlike T_2 and T_3. In T_2L_1 and T_3L_1, the coiled-coil density and part of the neck-linker from the leading head were low-pass filtered and displayed as a

mesh at a lower contour level than the main map. Both T_2L_1 and T_3L_1 represent two-heads-bound configurations, as evidenced by the densities of the coiled-coil and the neck-linkers positioned in opposite orientations. Notably, there is an increasing density of the inter-head connection (neck-linker and coiled-coil) from T_1L_1 to T_2L_1 to T_3L_1, in these classes, accounting for 1.3%, 11% and 10% of the particles in the MT-KIF1A-ANP dataset, respectively. The T_3L_1 class exhibits the strongest inter-head connection density (neck-linker and coiled-coil) in the entire MT-KIF1A-ANP dataset, despite having fewer particles than T_2L_1, suggesting a more rigid conformation than T_2L_1. In (**b**), the densities of T_1L_0, T_2L_0 and T_3L_0 are displayed, overlaid with low-pass filtered versions of these maps. These maps show a lack of kinesin density at site L, indicating that the motors detected on site T are in a one-head-bound configuration. In (**c**), each of the T_1L_*, T_2L_* and T_3L_* classes exhibit a docked neck-linker in both sites T and L, indicating that the motors detected on the site T are in a one-head-bound configuration, and that the motor seen on the site L corresponds to another kinesin dimer. **d-g:** Comparison of the conformations of the three detected KIF1A trailing heads, T_1, T_2 and T_3. For this comparison, maps of the one-head-bound-configuration T_1L_{0*}, and of the two-heads-bound configurations T_2L_1 and T_3L_1 were used. In (**d**), semi-transparent isosurfaces of the nucleotide-binding area of the kinesin are shown, with underlying models displayed with a cartoon representation. T_1, T_2 and T_3 models are overlaid with the same viewing angle in (**e**) and colored yellow, orange and red, respectively, as indicated. Additionally, the model of the leading head L_1 (from $T_{23}L_1$) is displayed in blue for comparison. T_1, T_2 and T_3 share the same H0 position, which is distinct from the open conformation found in L_1. Notably, the positions of H2 close to the nucleotide and loop-5 (L5) follow the order L_1, T_1, T_2, T_3, as indicated by the black arrow. This trend corresponds to a progressive closing of the nucleotide-binding pocket, with the nucleotide inserted further in the pocket. This gradient of conformation is particularly visible in the helices H1 and H2. Panels (**f-g**) provide a similar comparison as in (**d-e**), focusing on these two helices. Arrows on the model overlay in (**g**) emphasize the gradation of motor closing from L_1 to T_3, with T_2 and T_3 being the most similar among these four conformations. T_1 represents a semi-closed conformation (Fig. 2d, Supplementary Fig. 8) with both a docked neck-linker (**b-c**) and areas where it appears more similar to an open conformation (L_1) than T_2 and T_3, such as the location of helices H1, H2 and H3. Importantly, T_1 is a major conformation of the MT-bound head of KIF1A in the one-head-bound configuration:12% of the dataset in T_1 (T_1L_{0*}) vs. 4% for T_2 (T_2L_{0*}) and 6% for T_3 (T_2L_{0*}) are in one-head-bound configurations, as shown in Supplementary Fig. 3. However, it has only a very low representation in the two-heads-bound configuration (< 1.3% (T_1L_1) vs. 11% for T_2 (T_2L_1) and 10% for T_3 (T_3L_1). Therefore, these structural observations indicate that the binding of the leading head restricts the conformation of the trailing head to a more closed conformation (T_2 and T_3), which is presumably more favorable for ATP hydrolysis. This property of KIF1A, to have the dynamic nucleotide-binding pocket of the MT-bound head partially closed in the one-head-bound configuration and further closed once the leading head binds, is possibly related to its high processivity.

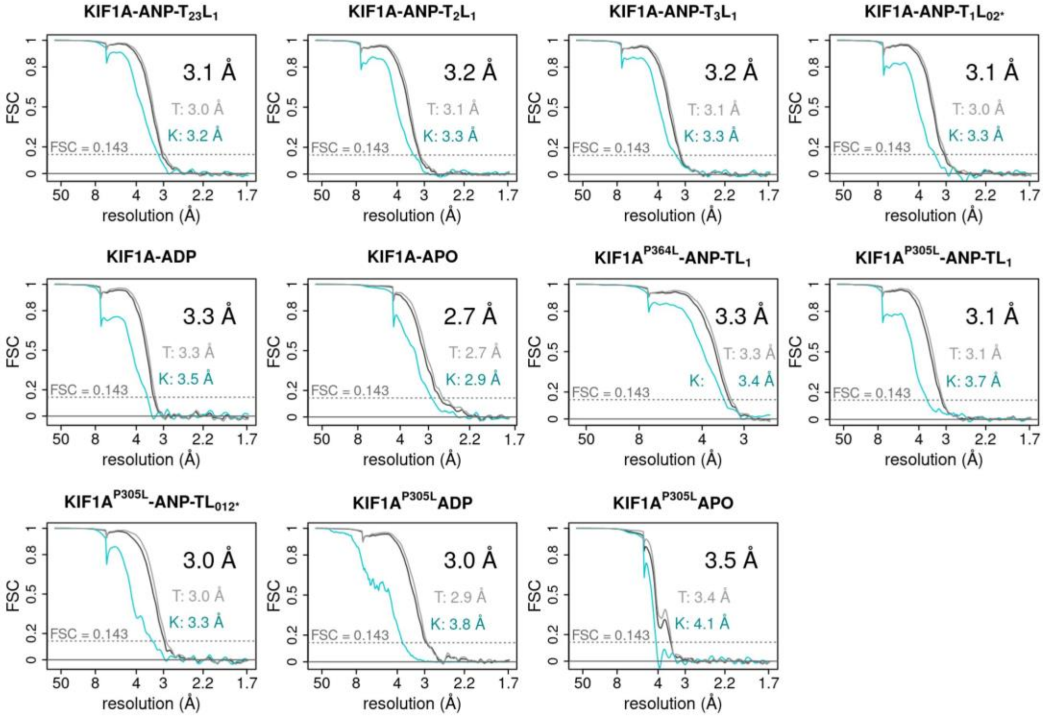

Supplementary Fig 5. Resolution estimation of the cryo-EM maps. The figure displays FSC curves for KIF1A structures determined by cryo-EM. The overall resolution is represented by the black curve, the resolution for the tubulin (T) component is shown in grey, and the resolution for the kinesin (K) component is depicted in turquoise. The resolution values ($FSC_{0.143}$) for the overall structure, the tubulin part, and the kinesin part are indicated. The half maps and masks used to generate the FSC curves have been deposited in the EMDB, with accession numbers provided in Supplementary Table 1.

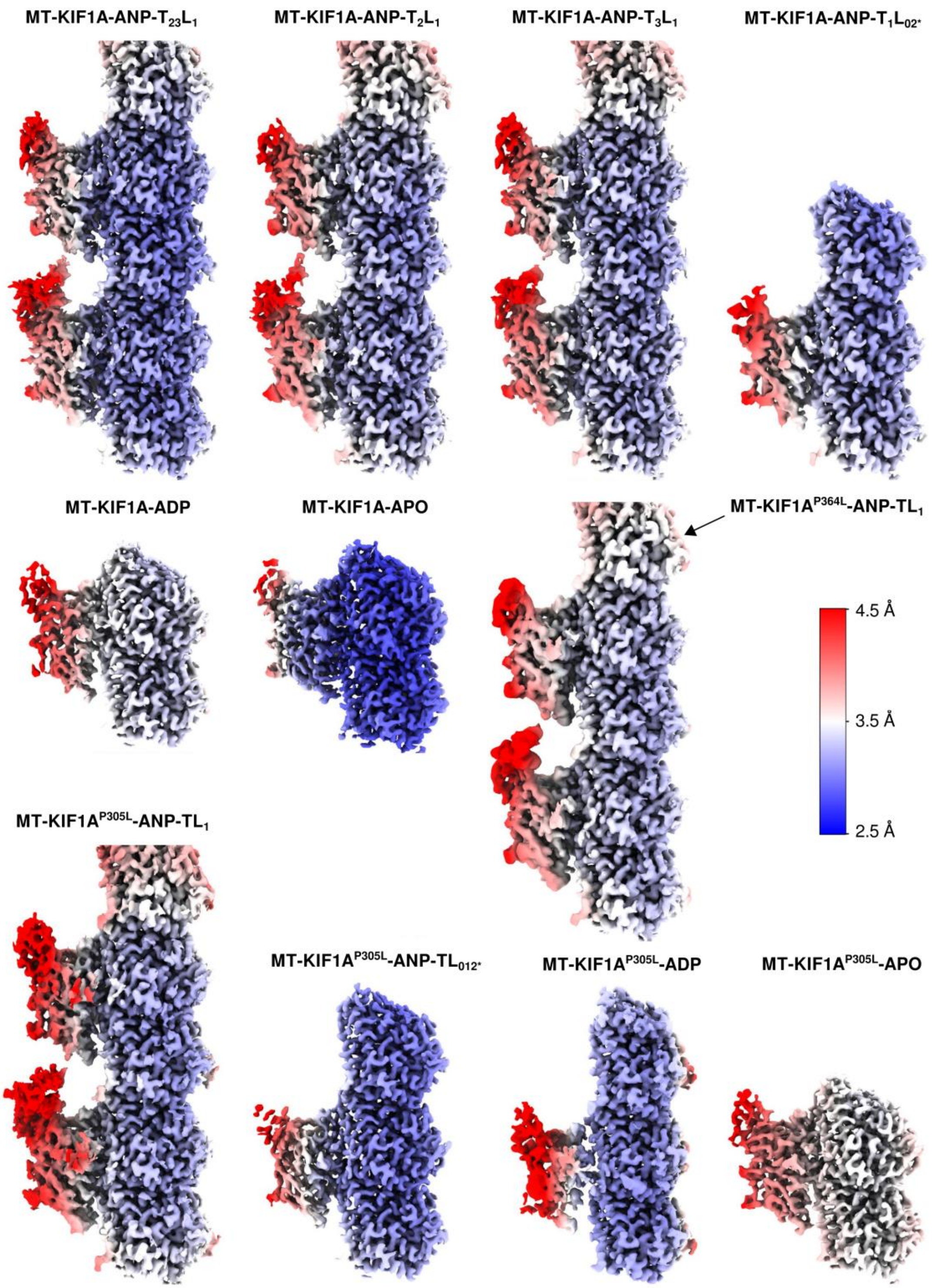

Supplementary Fig 6. Local Resolution. Isosurface representations of the cryo-EM maps colored according to the local resolution as indicated in the inset scale.

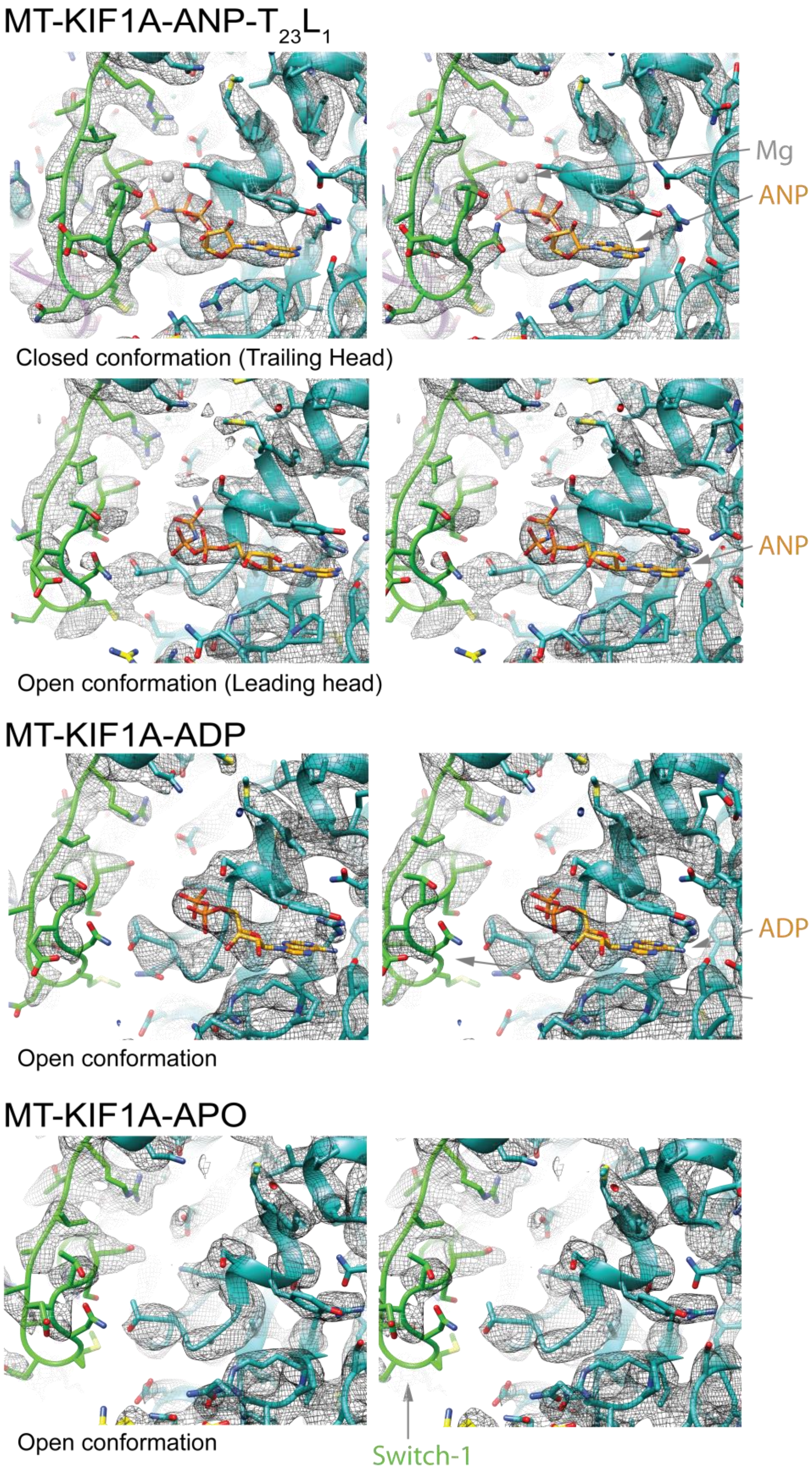

Supplementary Fig 7. KIF1A nucleotide pocket stereo-views. The figure shows the nucleotide binding pocket of the cryo-EM maps indicated by the top labels, MT-KIF1A-ANP-$T_{23}L_1$, MT-KIF1A-ADP and MT-KIF1A-APO. Cryo-EM map isosurfaces are shown with a grey mesh. Fitted atomic models are shown with the backbone in ribbon representation and the side chains as sticks. Kinesin backbone is colored blue with the switch-1 loop in green, the bound nucleotide in orange and the Mg cofactor as a light grey sphere.

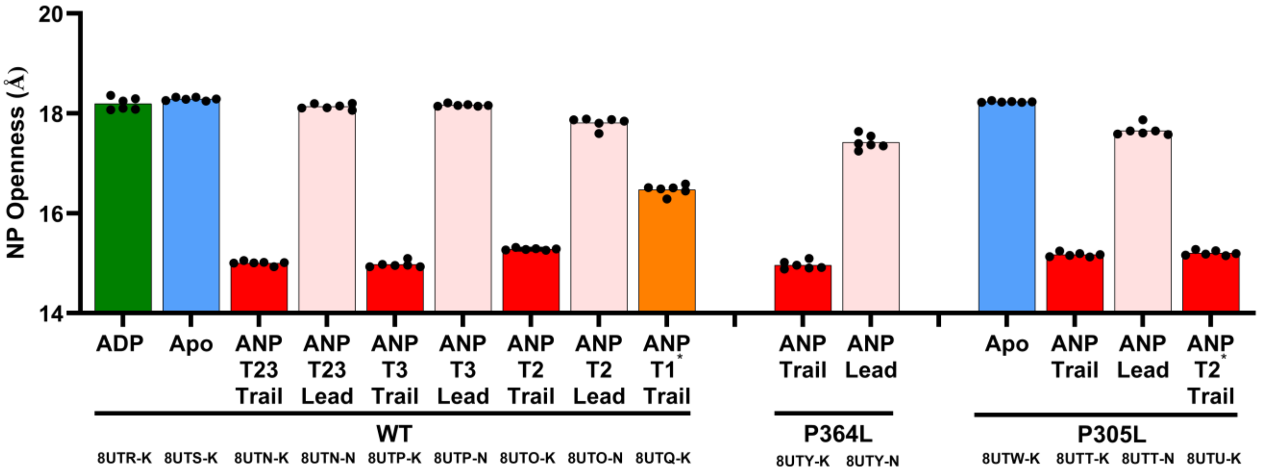

Supplementary Fig 8. Nucleotide Pocket (NP) Openness. NP openness was estimated as the average of six distances between Cα carbons of selected residues (R216 and A250 to P14, to S104, and to Y105) across the KIF1A nucleotide-binding pocket. Each column bar corresponds to an atomic model of the KIF1A motor domain. The PDB accession code and the chain of the KIF1A motor domain are indicated at the bottom of the figure (PDB accession code-chain). The NP openness was not calculated for the P305L-ADP model (8UTV-K) due to the lower resolution of the kinesin motor domain in the corresponding cryo-EM map (Supplementary Table 1), rendering this calculation unreliable. Column height corresponds to the mean of six openness values (black circles). These six openness values for each model correspond to the one calculated from the given model and the ones calculated from the best five of eighty regenerated Rosetta models (see methods).

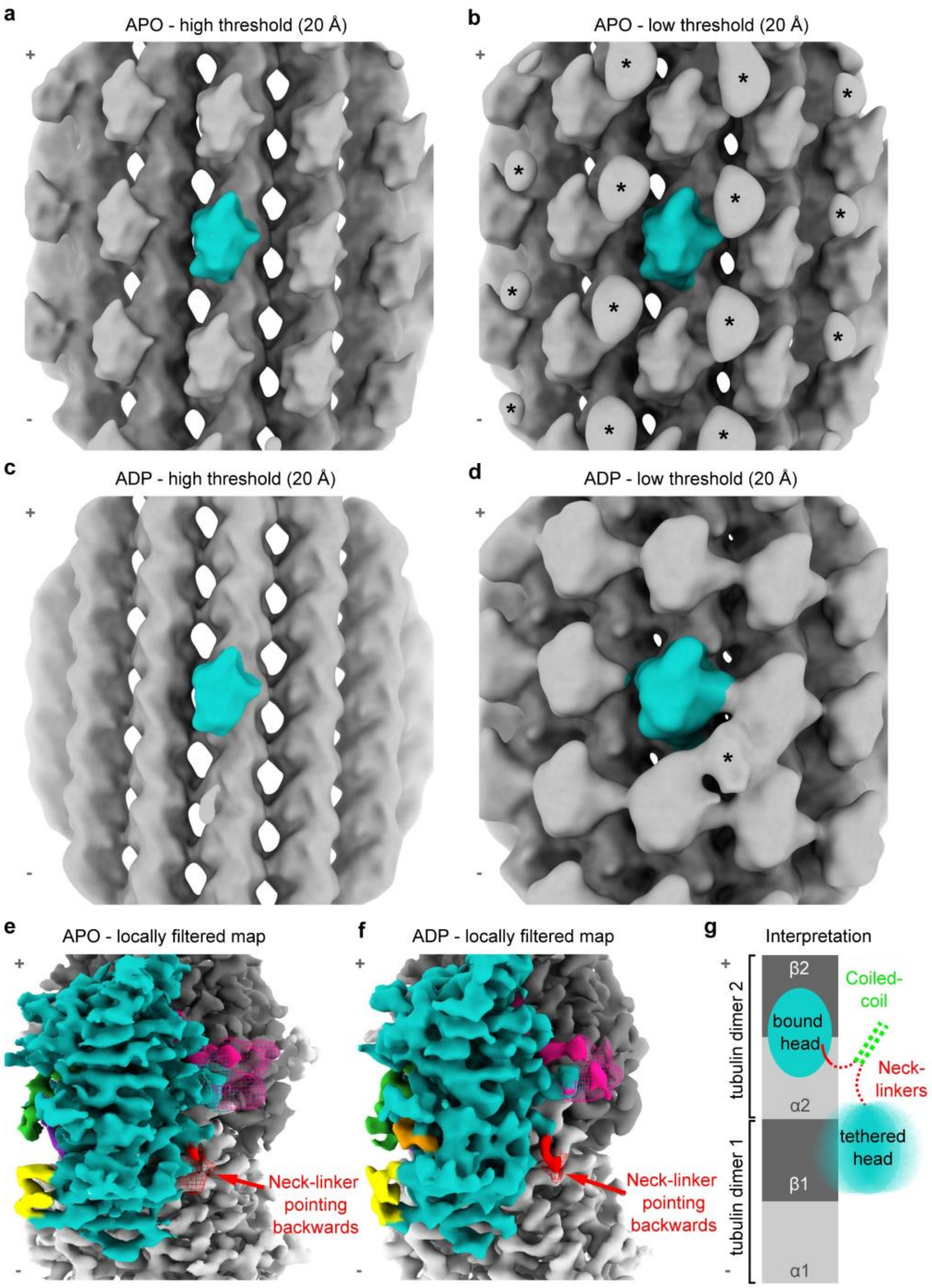

Supplementary Fig 9. Tethered Head Location. a-b: Shown are 20 Å low-passed filtered cryo-EM maps of WT KIF1A in the Apo state (MT-KIF1A-APO) at high and low threshold, respectively. The kinesin belonging to the central asymmetric unit, on which the refinement was focused, is colored blue. Notably, in (b), extra densities indicated with an asterisk (*) are visible between the MT-bound kinesin motors and away from the MT surface. Such densities were not observed in the ANP dataset. All the 20 Å low-passed filtered cryo-EM maps displayed in this figure have been deposited as additional maps in the corresponding EMDB entries. **c-d:** Similar to (**a-b**), but for WT KIF1A in the ADP state (MT-KIF1A-ADP). Due to the lower decoration (approximately 32%, as shown in Supplementary Table 2) of this dataset (**c**), only one extra density (*) is detected near the central kinesin motor (**d**). This suggests that this density is associated with the central motor and that the similar extra densities seen in (**b**) are also linked to the nearest motor on the (+) end of the same protofilament. These extra densities could correspond to the position of the mobile unbound head or possibly to the coiled-coil region. In either case, this indicates that the associated tethered head is positioned backwards. **e-f:** Isosurfaces of the maps of WT KIF1A in the Apo state (MT-KIF1A-APO) (**e**) and WT KIF1A in the ADP state (MT-KIF1A-ADP) (**f**). Densities for the weaker K-loop and neck-linker were low-passed filtered and are represented as a mesh. Note that in both cases, the first residues of the neck-linker are pointing backward, towards the (-) end of the MT. **g:** Proposed interpretation of the combined observations

in (**a-f**) for KIF1A in ADP and APO states. The resolved initial segment of the neck-linker and the extra low-resolution density present toward the (-) end of the motor suggests that the tethered head samples at least a position where it is oriented backward from the MT-bound motor.

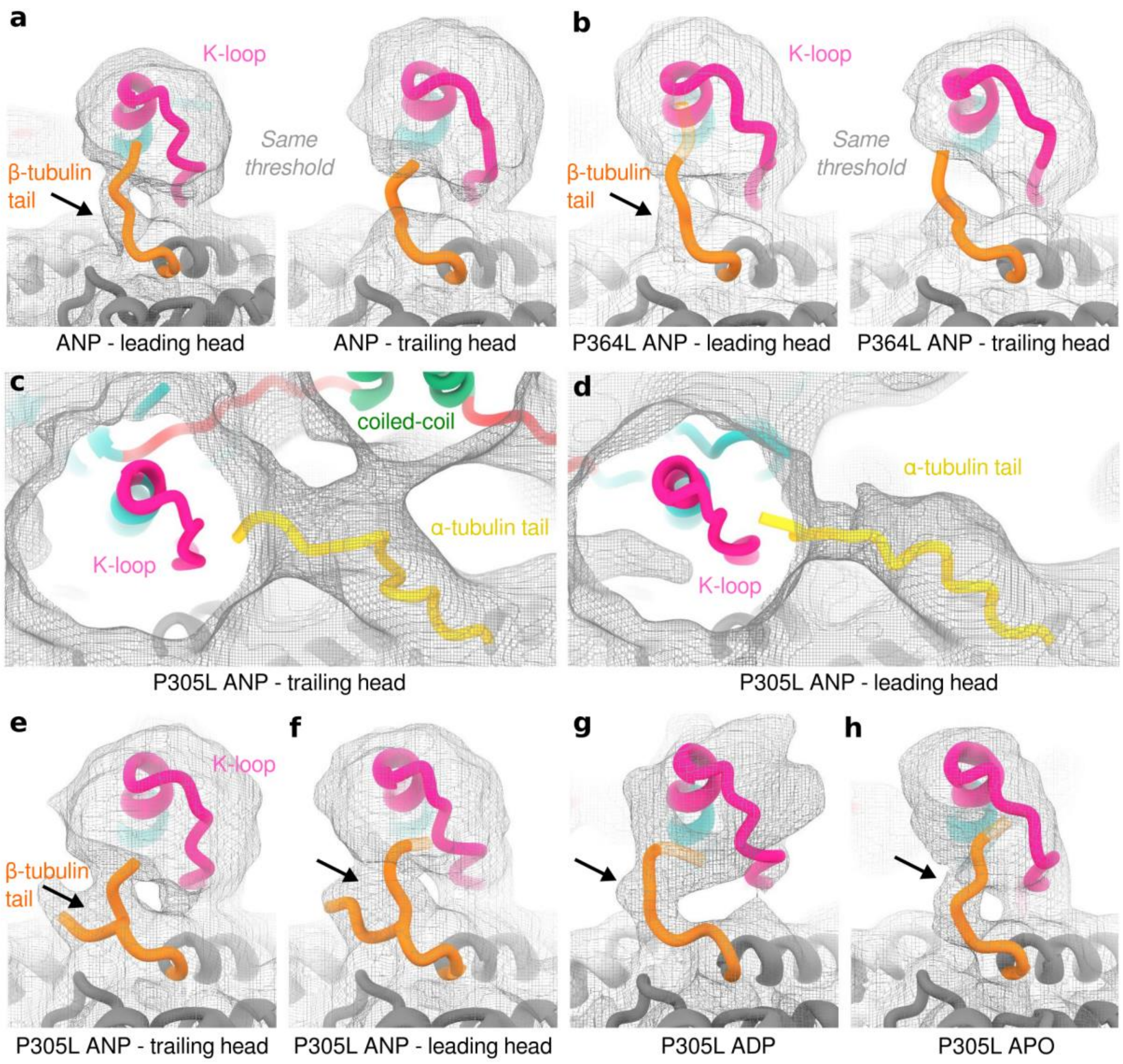

Supplementary Fig 10. Interaction of the K-loop with the C-terminal tubulin tails.

a: Shown is a 6 Å low-passed filtered cryo-EM map of the WT KIF1A two-heads-bound configuration in the ANP state (MT-KIF1A-ANP-$T_{23}L_1$), highlighting the areas around the β-tubulin tails near the leading and trailing heads of KIF1A, displayed at the same threshold. Notably, the density assigned to the β-tubulin tail is more resolved for the tail near the leading head than for the one near the trailing head of KIF1A. **b:** Similar to (**a**) but for the KIF1A P364L mutant two-heads-bound configuration in the ANP state (MT-KIF1A^{P364L}-ANP-TL_1). **c-d:** Displayed are 8 Å low-passed filtered cryo-EM map of the two-heads-bound configuration of the KIF1A P305L mutant (KIF1A^{P305L}-ANP-TL_1), showing densities of α-tubulin C-terminal tails reaching the K-loops of the trailing (**c**) or leading (**d**) head. Similar to WT KIF1A (Fig. 5b, c), the α-tubulin tail interacting with the trailing head is located within a pocket of positively-charged residues due to the charges of K-loop, the nearby coiled-coil, and the docked neck-linker. Given the shape of the map in this area, it is likely that additional interactions between the α-tubulin tail and the KIF1A coiled-coil occur. **e-h:** Presented are 6 Å low-passed filtered maps of KIF1A near the K-loop in distinct nucleotide states and motor-domain conformations. The density associated with the β-tubulin C-terminal tail is indicated with an arrow, and the threshold was adjusted for each map. It is worth noting that in the ANP state, the β-tubulin tail density is better resolved in the leading head than in the trailing head. The 6 Å and 8Å low-passed filtered maps used in this figure have been deposited as additional maps in the corresponding EMDB entries.

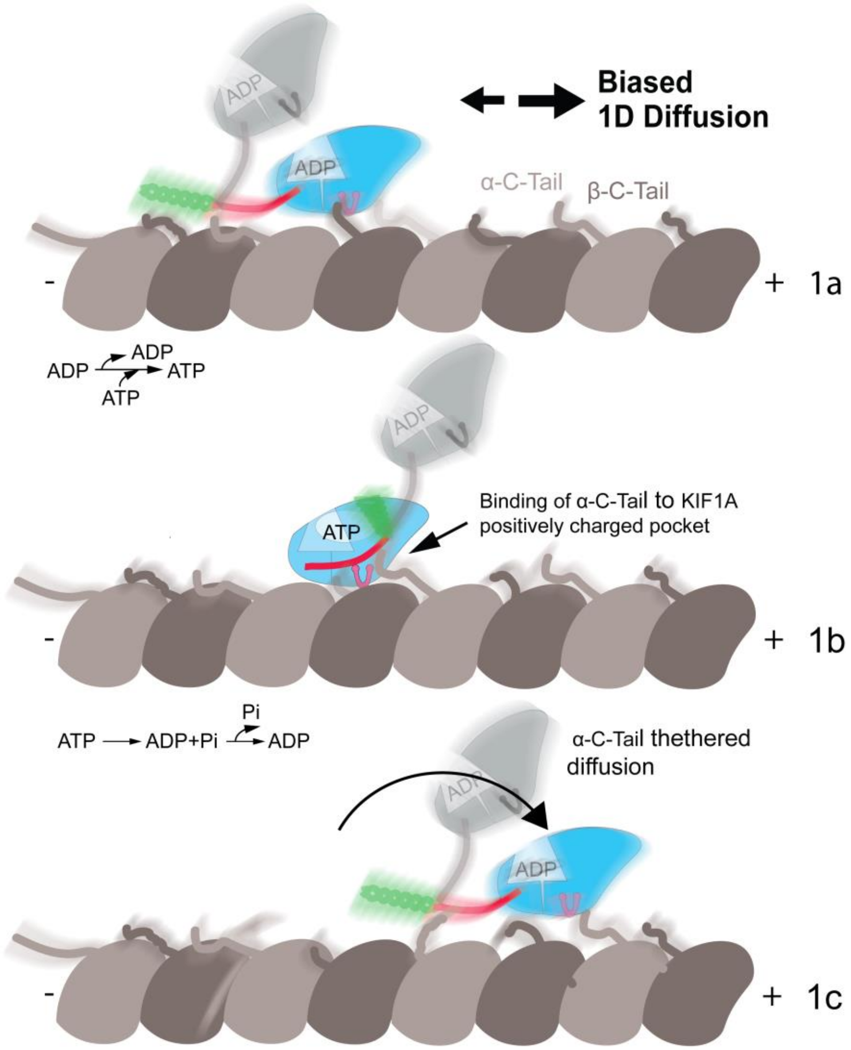

Supplementary Fig. 11. Biased diffusion model. The figure shows a hypothetical model for KIF1A's MT-plus-end-biased one-dimensional-diffusion, inferred from the cryo-EM KIF1A-MT complex structures. In the ADP state, electrostatic interactions between the KIF1A K-loop and the tubulin C terminal tails (Fig. 5b) allow KIF1A to weakly attach to the MT, enabling unbiased diffusion along the MT (step 1a). In this state, one or both KIF1A heads may weakly interact with the MT (a single-head interaction is shown in this figure). Strong attachment of one motor head triggers nucleotide pocket opening and ADP release. Subsequently, ATP binding to the open nucleotide binding pocket induces neck-linker docking (step 1b). In this step, the docked neck-linker, together with the K-loop and part of the coiled-coil domain, creates a pocket of positively charged residues (Fig. 5b). This pocket promotes interaction with the C-tail of α-tubulin, which is located at the MT plus-end relative to the bound motor domain. Completing one ATP-hydrolysis cycle and returning to the ADP state causes the MT-attached head to exit the strongly-bound state, and the neck-linker to undock. Undocking of the neck-linker and the weak attachment to the α-tubulin C-tails results in a step in the MT-plus-direction (step 1c). Combining unbiased one-dimensional diffusion (1b) with MT-plus-end-directed steps (1b-c) results in MT-plus-end-biased one dimensional-diffusion. The proposed model can operate with the single head of a KIF1A monomer. A dimeric KIF1A molecule would have the ability to combine hand-over-hand motion (Fig. 7) with MT-plus-end-biased motility, minimizing the chances of MT detachment during runs and contributing to the characteristic high processivity of KIF1A.

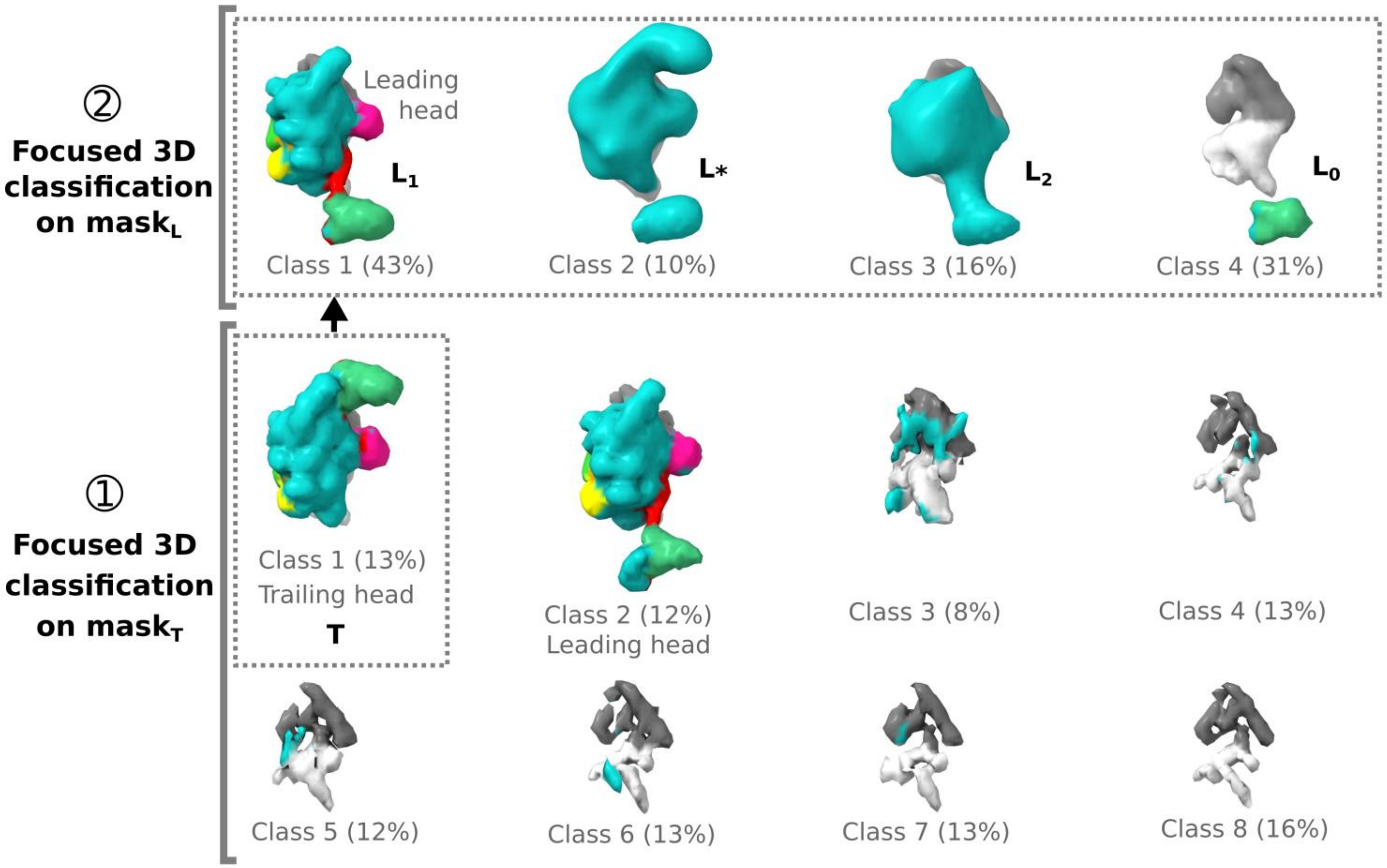

Supplementary Fig 12. 3D classification strategy used for the MT-KIF1A^{P305L}-ANP dataset. The same 3D classification strategy employed for the MT-KIF1A-ANP dataset was applied to the MT-KIF1A^{P305L}-ANP dataset. The first 3D classification in eight classes focusing on mask$_T$ (step ①) led to the eight class averages displayed in the lower part of the figure. Class 1 corresponds to kinesin motors with the coiled-coil density detected toward the (+) end of the motor (forward), while class 2 corresponds to a leading head with the undocked neck-linker connected to a coiled-coil present toward the (-) end of the motor domain (backward). The other six classes appear undecorated by a kinesin. The single class with a closed conformation was named 'class T' and following the same strategy as in the MT-KIF1A-ANP dataset, it was further classified in step ② in four classes on the site L (with mask$_L$). The corresponding four class averages are displayed in the upper part of the figure. The major class (class 1) corresponds to a motor in the leading head conformation with an undocked neck-linker pulled backward and connected to a coiled-coil whose density is visible. The class TL$_1$ therefore corresponds to a two-heads-bound kinesin configuration. Classes 2 and 3, observed at a lower resolution, display similarities with a trailing and leading head, respectively. These were named L$_*$ and L$_2$. However, due to their lower resolution these classes could not be confidently assigned to these specific states, unlike class L$_1$, which exhibited distinct features. Class 4 is devoid of kinesin associated densities so the corresponding motor domains on the site T (class TL$_0$) correspond to a one-head-bound configuration, with no leading head bound in the forward direction.

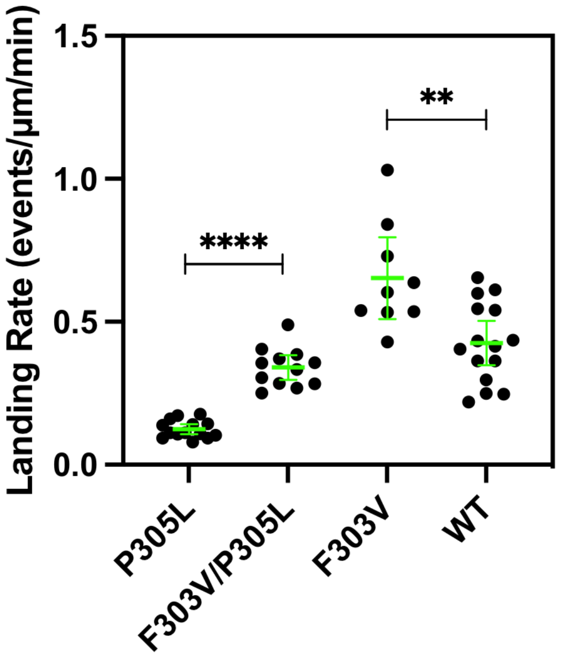

Supplementary Fig 13. Landing rates. The landing rates of P305L, F303V/P305L, F303V, and WT. The green bars represent the mean with 95% confidence interval. P305L: 0.12 [0.11, 0.14] events/μm MT/min; F303V/P305L: 0.34 [0.30, 0.38] events/μm MT/min; F303V: 0.65 [0.51, 0.80] events/μm MT/min; WT: 0.43 [0.35, 0.50] events/μm MT/min. The statistics were performed using unpaired t-test (**: P<0.01; ****: P<0.0001).

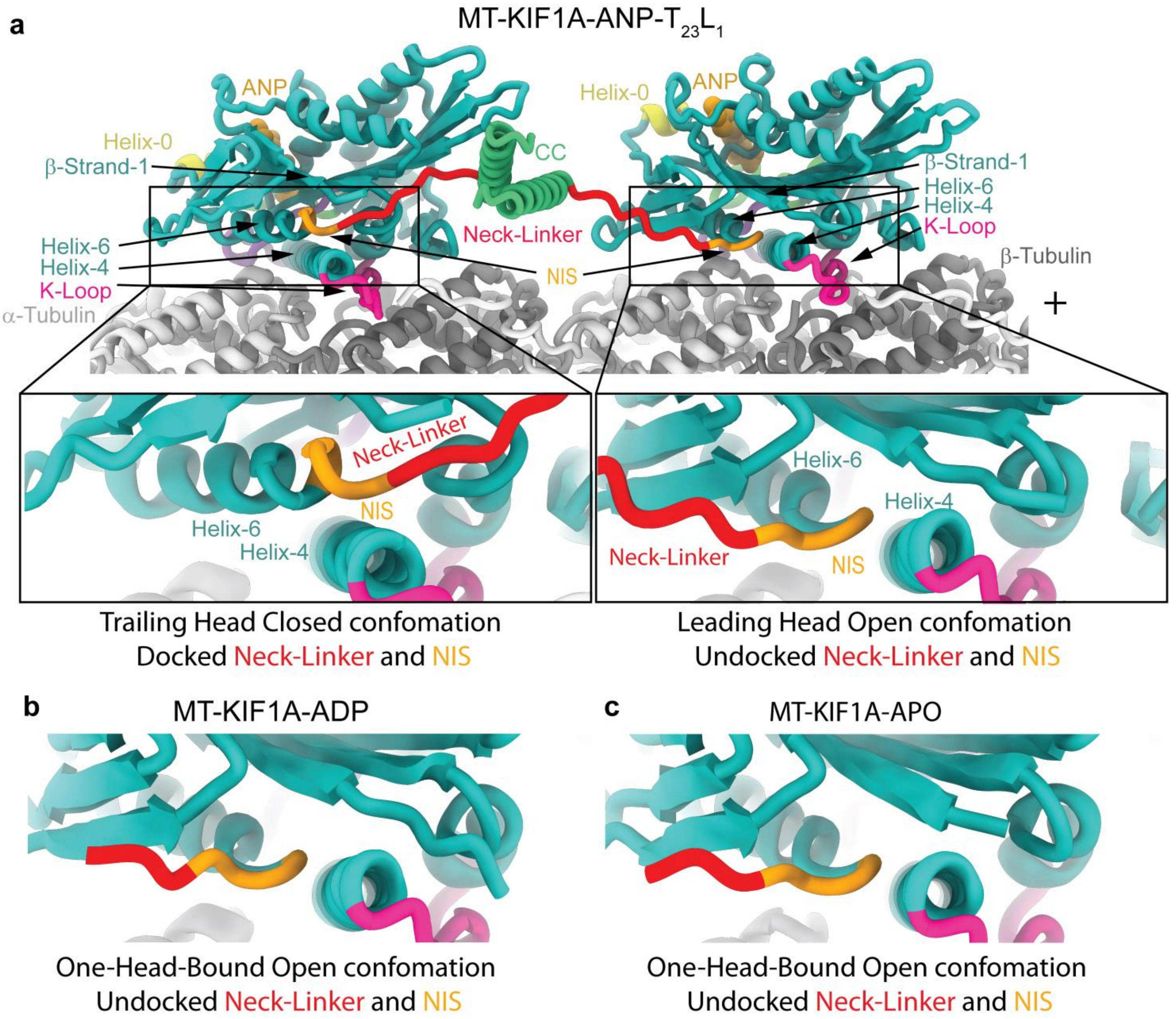

Supplementary Fig 14. KIF1A Neck Linker (NL) and Neck-Linker-Initial-Segment (NIS) in the Open and Closed conformations. a: MT-KIF1A-ANP two-heads-bound state model. The top panel shows the full kinesin-motor domains and the bottom panels a close-up of the NIS-NL regions of the trailing (left) and leading (right) head. **b-c:** NIS-NL region close-up of MT-KIF1A-ADP (b) and MT-KIF1A-APO (c) one-head bound models. Note that the NIS in the closed conformation (trailing head, two-heads-bound structure) forms the last turn of helix-6 and the start of the neck-linker is docked in the pocket formed between helix-4 and β-strand-1. In the open conformation observed in the leading head of the two head bound ANP structure and in the ADP and APO one-head-bound structures the NIS and NL are undocked and pointing backwards (towards the MT minus end). NIS is colored ochre and other structure parts colored as indicated as in Figs. 1 and 2.

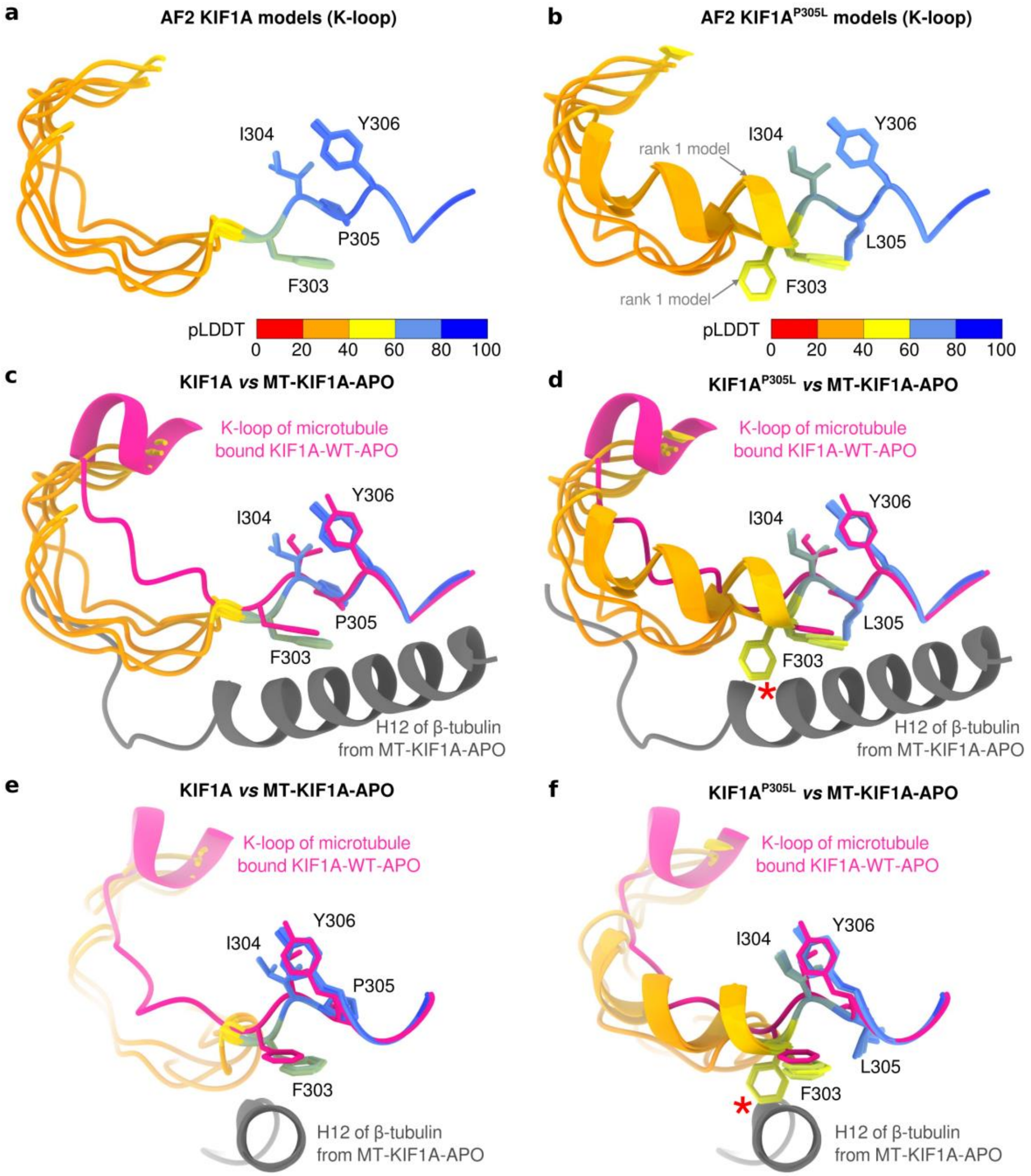

Supplementary Fig. 15. Comparison of AlphaFold2 models of WT KIF1A vs. KIF1A^{P305L} K-loops when not interacting with the MT. a: Overlay of the 5 best models of the K-loops from a WT KIF1A motor, generated using the ColabFold[85] implementation of AlphaFold2 (AF2)[86]. The models were aligned based on residues 304 to 310. Residues are color-coded according to their confidence scores (pLDDT). Note that the structural models of the K-loop are very similar. **b:** Same as (**a**), but for the KIF1A P305L mutant. Notably, the models exhibit greater variability compared to those of WT KIF1A in (**a**). The highest-ranked model is indicated by two arrows. This model together with another model, features a different configuration of F303 relative to WT as well as differences in secondary structure in the lower confidence regions of the models that include the more mobile portion of the K-loop containing lysines. **c-d:** Overlays of the same structures as in (**a**) and (**b**) compared with the experimental MT-KIF1A-APO structure. The alignment is performed using the same residues as in (**a-b**). These comparisons were made to assess the local structural changes required for MT binding. In the case of the KIF1A P305L mutant, the alternate conformation of F303 mentioned in (**b**) would clash with helix H12 of β-tubulin (indicated by an asterisk), which is unfavorable for MT binding, as observed. **e-f:** The same alignments as in (c-d), viewed along the axis of helix H12 of β-tubulin from the (+) to the (-) end of the MT.

Supplementary Table 1. Cryo-EM data collection, refinement and validation statistics (1/3)

	**MT-KIF1A-ANP-$T_{23}L_1$** (EMDB-42543) (PDB 8UTN)	**MT-KIF1A-ANP-T_2L_1** (EMDB-42544) (PDB 8UTO)	**MT-KIF1A-ANP-T_3L_1** (EMDB-42545) (PDB 8UTP)	**MT-KIF1A-ANP-T_1L_{02*}** (EMDB-42546) (PDB 8UTQ)
**Data collection and processing**				
Magnification (actual)	59242	59242	59242	59242
Voltage (kV)	300	300	300	300
Electron exposure ($e^–/Å^2$)	50.2	50.2	50.2	50.2
Defocus range (μm) [a]	0.8 ; 1.7	0.8 ; 1.7	0.8 ; 1.7	0.8 ; 1.7
Pixel size (Å)	0.844	0.844	0.844	0.844
Symmetry imposed [b]	Helical	Helical	Helical	Helical
Rise (Å)	5.60	5.60	5.60	5.60
Twist (deg)	168.09	168.09	168.09	168.09
Particle images identified as 15R symmetry (no.)	60199	60199	60199	60199
Particle images in helical reconstruction (no.)	60199	60199	60199	60199
Single particles (no.) [c]	902985	902985	902985	902985
Single particles used (no.) [d]	189036	99325	89711	116194
Overall resolution (Å)	3.1	3.2	3.2	3.1
FSC threshold	0.143	0.143	0.143	0.143
Kinesin resolution (Å)	3.2	3.3	3.3	3.3
Tubulin resolution (Å)	3.0	3.1	3.1	3.0
**Refinement**				
Model composition				
Non-hydrogen atoms	23787	23792	23792	13356
Protein residues	2981	2982	2982	1680
Ligands	13	13	13	8
R.m.s. deviations				
Bond lengths (Å)	0.0063	0.0053	0.0054	0.0053
Bond angles (°)	1.18	1.12	1.13	1.13
Validation				
MolProbity score	1.73	1.45	1.52	1.51
Clashscore	4.92	3.70	4.14	3.99
Poor rotamers (%)	1.64	0.59	0.94	0.28
Ramachandran plot				
Favored (%)	95.48	95.86	95.35	95.39
Allowed (%)	3.67	3.84	4.21	4.49
Disallowed (%)	0.84	0.30	0.44	0.12

[a] Range of the per-particle defocus estimates. This range encompasses the defocus values for 90% of the particles used, with 5% of the particle defocuses falling below and 5% exceeding this range.
[b] Symmetry is imposed during the initial symmetrical refinement preceding the local refinements.
[c] Total number of particles after symmetry expansion.
[d] Number of asymmetric units used after 3D classification, corresponding to a kinesin motor bound to a tubulin dimer.

Supplementary Table 1. Cryo-EM data collection, refinement and validation statistics (2/3)

	**MT-KIF1A-ADP** (EMDB-42547) (PDB 8UTR)	**MT-KIF1A-APO** (EMDB-42548) (PDB 8UTS)	**MT-KIF1A^{P364L}-ANP-TL$_1$** (EMDB-42553) (PDB 8UTY)	**MT-KIF1A^{P305L}-ANP-TL$_1$** (EMDB-42549) (PDB 8UTT)
**Data collection and processing**				
Magnification (actual)	59242	57078	59242	59242
Voltage (kV)	300	300	300	300
Electron exposure (e^-/Å^2)	67.5	62.9	57.5	49.4
Defocus range (μm) [a]	0.9 ; 2.1	0.7 ; 1.7	1.2 ; 2.8	1.0. ; 2.4
Pixel size (Å)	0.844	0.876	1.3	0.844
Symmetry imposed [b]	Helical	Helical	Helical	Helical
Rise (Å)	5.61	5.61	5.58	5.64
Twist (deg)	168.09	168.09	168.09	168.09
Particle images identified as 15R symmetry (no.)	24372	52581	74962	125182
Particle images in helical reconstruction (no.)	24372	52581	74962	125182
Single particles (no.) [c]	365580	788715	1124430	1877730
Single particles used (no.) [d]	118273	685161	137556	110600
Overall resolution (Å)	3.3	2.7	3.3	3.1
FSC threshold	0.143	0.143	0.143	0.143
Kinesin resolution (Å)	3.5	2.9	3.4	3.7
Tubulin resolution (Å)	3.3	2.7	3.3	3.1
**Refinement**				
Model composition				
Non-hydrogen atoms	9793	9749	23765	23745
Protein residues	1230	1227	2979	2974
Ligands	5	4	13	13
R.m.s. deviations				
Bond lengths (Å)	0.0057	0.0055	0.0059	0.0075
Bond angles (°)	1.16	1.17	1.16	1.23
Validation				
MolProbity score	1.47	1.96	1.75	1.91
Clashscore	3.73	4.69	5.02	6.57
Poor rotamers (%)	0.66	2.56	1.45	1.60
Ramachandran plot				
Favored (%)	95.51	93.78	94.81	94.26
Allowed (%)	4.33	5.41	4.38	5.30
Disallowed (%)	0.16	0.82	0.81	0.44

[a] Range of the per-particle defocus estimates. This range encompasses the defocus values for 90% of the particles used, with 5% of the particle defocuses falling below and 5% exceeding this range.
[b] Symmetry is imposed during the initial symmetrical refinement preceding the local refinements.
[c] Total number of particles after symmetry expansion.
[d] Number of asymmetric units after 3D classification, corresponding to a kinesin motor bound to a tubulin dimer.

Supplementary Table 1. Cryo-EM data collection, refinement and validation statistics (3/3)

	MT-KIF1A^{P305L} -ANP-TL$_{012*}$ (EMDB-42550) (PDB 8UTU)	MT-KIF1A^{P305L} -ADP (EMDB-42551) (PDB 8UTV)	MT-KIF1A^{P305L} -APO (EMDB-42552) (PDB 8UTW)
**Data collection and processing**			
Magnification (actual)	59242	57078	59242
Voltage (kV)	300	300	300
Electron exposure (e$^-$/Å^2)	49.4	62.9	50.4
Defocus range (µm) [a]	1.0 ; 2.4	0.8 ; 2.0	1.2. ; 2.2
Pixel size (Å)	0.844	0.876	0.844
Symmetry imposed [b]	Helical	Helical	Helical
Rise (Å)	5.64	5.64	5.62
Twist (deg)	168.09	168.09	168.09
Particle images identified as 15R symmetry (no.)	125182	83861	79145
Particle images in helical reconstruction (no.)	125182	83861	79145
Single particles (no.) [c]	1877730	1257915	1187175
Single particles used (no.) [d]	254197	119590	620149
Overall resolution (Å)	3.0	3.0	3.5
FSC threshold	0.143	0.143	0.143
Kinesin resolution (Å)	3.3	3.8[e]	4.1
Tubulin resolution (Å)	3.0	2.9	3.4
**Refinement**			
Model composition			
Non-hydrogen atoms	13615	13338	9787
Protein residues	1709	1679	1227
Ligands	8	7	4
R.m.s. deviations			
Bond lengths (Å)	0.0064	0.0058	0.0056
Bond angles (°)	1.23	1.18	1.16
Validation			
MolProbity score	1.68	1.53	1.97
Clashscore	5.23	4.76	5.96
Poor rotamers (%)	1.50	0.70	1.89
Ramachandran plot			
Favored (%)	96.06	95.87	93.28
Allowed (%)	3.70	4.07	6.47
Disallowed (%)	0.24	0.06	0.25

[a] Range of the per-particle defocus estimates. This range encompasses the defocus values for 90% of the particles used, with 5% of the particle defocuses falling below and 5% exceeding this range.
[b] Symmetry is imposed during the initial symmetrical refinement preceding the local refinements.
[c] Total number of particles after symmetry expansion.
[d] Number of asymmetric units after 3D classification, corresponding to a kinesin motor bound to a tubulin dimer.
[e] Note that in this map kinesin density is weak and of low-resolution with no side-chains resolved unlike in the tubulin part of the maps. As a consequence, the kinesin resolution given is an overestimate dominated by the signal near the microtubule interface (see Supplementary Fig. 6).

Dataset	Kinesin concentration (μM)	Buffer	Decorated (%)	Undecorated (%)	Unknown (%)
MT-KIF1A-ANP	20	BRB80	74	21	5
MT-KIF1A-ADP	40	BRB80	32	32	36
MT-KIF1A-APO	40	BRB80	95	0	5
MT-KIF1A^{P305L}-ANP	40	BRB36	25	75	0
MT-KIF1A^{P305L}-ADP	40	BRB36	9	86	5
MT-KIF1A^{P305L}-APO	40	BRB36	91	9	0

Supplementary Table 2. Levels of decoration in each of the cryo-EM datasets. The decorated fraction corresponds to the proportion of the particles images assigned to class(es) for which the class average(s) after the focused 3D classification on the single kinesin site (or site T for ANP datasets) shows a density that could be recognized as being a kinesin motor domain bound to the tubulin dimer. The undecorated fraction corresponds to class averages showing a lack of kinesin motor domain present on the tubulin dimer. In most datasets, there are some low-resolution classes (like Class 6 in Supplementary Fig. 3) for which the class averages show a density that was not reliably assigned as decorated or undecorated and such cases are listed as unknown in the table. Kinesin concentrations shown in the second column correspond to the concentration of KIF1A monomers (single polypeptide).

Formula and datasets	*f* (2HB) (%)	*f* (1HB) (%)	*f** (2HB) (%)	*f** (1HB) (%)
Formula	$\frac{100 \times n(\mathrm{2HB})}{n(\mathrm{1HB_{all}}) + n(\mathrm{2HB})}$	$\frac{100 \times n(\mathrm{1HB_{all}})}{n(\mathrm{1HB_{all}}) + n(\mathrm{2HB})}$	$\frac{100 \times n(\mathrm{2HB})}{n(\mathrm{1HB_{free}}) + n(\mathrm{2HB})}$	$\frac{100 \times n(\mathrm{1HB_{free}})}{n(\mathrm{1HB_{free}}) + n(\mathrm{2HB})}$
MT-KIF1A-ANP	36	64	67	33
MT-KIF1A^{P305L}-ANP	58	42	58	42
MT-KIF1A^{P364L}-ANP	38	62	49	51

Supplementary Table 3. Relative frequency of two-heads and one-head bound configurations in the ANP datasets. The relative abundance of the two-heads-bound configurations (2HB) and of all the one-head-bound configurations ($1HB_{all}$) are provided in the first two columns and named *f*(2HB) and *f*(1HB) respectively. However, because of the molecular crowding on the microtubule, some of the observed one-head-bound-configurations - not all - are due to the fact two consecutive tubulin dimer were not available for binding a two-heads-bound configuration. This is shown in Supplementary Fig 4c where 2 motors in a closed state are bound consecutively (to compare with Supplementary Fig 4b showing bona fide one-heads-bound configurations). The number of two-heads-bound configurations is therefore underestimated with *f*(2HB). It is not possible to know exactly how many more two-heads-bound state would be seen if the decoration of the dataset would be much sparser. However, an estimate can be provided by excluding from the count the one-head-bound state that are clearly in a crowded area (as in Supplementary Fig 4c), keeping only the unambiguous ones ($1HB_{free}$) like in Supplementary Fig 4b. This is what *f**(2HB) and *f**(1HB) provide. Classes with associated class averages at low resolution and/or for which the one-head-bound or two-heads-bound configuration status was unclear were not included in these estimates.
