# Supplementary material for: Cryo-EM unveils kinesin KIF1A’s processivity mechanism and the impact of its pathogenic variant P305L
Source: Nat Commun. 2024 Jul 2;15:5530. doi: 10.1038/s41467-024-48720-4 (PMC11219953; doi:10.1038/s41467-024-48720-4)
Supplement: Supplementary file 4 — Source Data [file 41467_2024_48720_MOESM4_ESM.zip › Source_Data/Supplementary_Table_1.docx]

**Supplementary Table 1. Cryo-EM data collection, refinement and validation statistics (1/3)**

|  | **MT-KIF1A-ANP-T_23_L_1_**  (EMDB-42543) (PDB 8UTN) | **MT-KIF1A-ANP-T_2_L_1_**  (EMDB-42544)  (PDB 8UTO) | | **MT-KIF1A-ANP-T_3_L_1_**  (EMDB-42545)  (PDB 8UTP) | | **MT-KIF1A-ANP-T_1_L_02*_**  (EMDB-42546)  (PDB 8UTQ) |
| --- | --- | --- | --- | --- | --- | --- |
| **Data collection and processing** |  | |  |  |  | |
| Magnification (actual) | 59242 | | 59242 | 59242 | 59242 | |
| Voltage (kV) | 300 | | 300 | 300 | 300 | |
| Electron exposure (e^–^/Å^2^) | 50.2 | | 50.2 | 50.2 | 50.2 | |
| Defocus range (μm) ^a^ | 0.8 ; 1.7 | | 0.8 ; 1.7 | 0.8 ; 1.7 | 0.8 ; 1.7 | |
| Pixel size (Å) | 0.844 | | 0.844 | 0.844 | 0.844 | |
| Symmetry imposed ^b^  Rise (Å)  Twist (deg) | Helical  5.60  168.09 | | Helical  5.60  168.09 | Helical  5.60  168.09 | Helical  5.60  168.09 | |
| Particle images identified as 15R symmetry (no.) | 60199 | | 60199 | 60199 | 60199 | |
| Particle images in helical reconstruction (no.) | 60199 | | 60199 | 60199 | 60199 | |
| Single particles (no.) ^c^ | 902985 | | 902985 | 902985 | 902985 | |
| Single particles used (no.) ^d^ | 189036 | | 99325 | 89711 | 116194 | |
| Overall resolution (Å)  FSC threshold | 3.1  0.143 | | 3.2  0.143 | 3.2  0.143 | 3.1  0.143 | |
| Kinesin resolution (Å) | 3.2 | | 3.3 | 3.3 | 3.3 | |
| Tubulin resolution (Å) | 3.0 | | 3.1 | 3.1 | 3.0 | |
|  |  | |  |  |  | |
| **Refinement** |  | |  |  |  | |
| Model composition  Non-hydrogen atoms  Protein residues  Ligands | 23787  2981  13 | | 23792  2982  13 | 23792  2982  13 | 13356  1680  8 | |
| R.m.s. deviations  Bond lengths (Å)  Bond angles (°) | 0.0063  1.18 | | 0.0053  1.12 | 0.0054  1.13 | 0.0053  1.13 | |
| Validation  MolProbity score  Clashscore  Poor rotamers (%) | 1.73  4.92  1.64 | | 1.45  3.70  0.59 | 1.52  4.14  0.94 | 1.51  3.99  0.28 | |
| Ramachandran plot  Favored (%)  Allowed (%)  Disallowed (%) | 95.48  3.67  0.84 | | 95.86  3.84  0.30 | 95.35  4.21  0.44 | 95.39  4.49  0.12 | |

^a^ Range of the per-particle defocus estimates. This range encompasses the defocus values for 90% of the particles used, with 5% of the particle defocuses falling below and 5% exceeding this range.

^b^ Symmetry is imposed during the initial symmetrical refinement preceding the local refinements.

^c^ Total number of particles after symmetry expansion.

^d^ Number of asymmetric units used after 3D classification, corresponding to a kinesin motor bound to a tubulin dimer.

**Supplementary Table 1. Cryo-EM data collection, refinement and validation statistics (2/3)**

|  | **MT-KIF1A-ADP**  (EMDB-42547)  (PDB 8UTR) | **MT­-KIF1A-APO**  (EMDB-42548)  (PDB 8UTS) | **MT-KIF1A^P364L^**  **-ANP-TL_1_**  (EMDB-42553)  (PDB 8UTY) | **MT-KIF1A^P305L^**  **-ANP-TL_1_**  (EMDB-42549)  (PDB 8UTT) |
| --- | --- | --- | --- | --- |
| **Data collection and processing** |  |  |  |  |
| Magnification (actual) | 59242 | 57078 | 59242 | 59242 |
| Voltage (kV) | 300 | 300 | 300 | 300 |
| Electron exposure (e^–^/Å^2^) | 67.5 | 62.9 | 57.5 | 49.4 |
| Defocus range (μm) ^a^ | 0.9 ; 2.1 | 0.7 ; 1.7 | 1.2 ; 2.8 | 1.0. ; 2.4 |
| Pixel size (Å) | 0.844 | 0.876 | 1.3 | 0.844 |
| Symmetry imposed ^b^  Rise (Å)  Twist (deg) | Helical  5.61  168.09 | Helical  5.61  168.09 | Helical  5.58  168.09 | Helical  5.64  168.09 |
| Particle images identified as 15R symmetry (no.) | 24372 | 52581 | 74962 | 125182 |
| Particle images in helical reconstruction (no.) | 24372 | 52581 | 74962 | 125182 |
| Single particles (no.) ^c^ | 365580 | 788715 | 1124430 | 1877730 |
| Single particles used (no.) ^d^ | 118273 | 685161 | 137556 | 110600 |
| Overall resolution (Å)  FSC threshold | 3.3  0.143 | 2.7  0.143 | 3.3  0.143 | 3.1  0.143 |
| Kinesin resolution (Å) | 3.5 | 2.9 | 3.4 | 3.7 |
| Tubulin resolution (Å) | 3.3 | 2.7 | 3.3 | 3.1 |
|  |  |  |  |  |
| **Refinement** |  |  |  |  |
| Model composition  Non-hydrogen atoms  Protein residues  Ligands | 9793  1230  5 | 9749  1227  4 | 23765  2979  13 | 23745  2974  13 |
| R.m.s. deviations  Bond lengths (Å)  Bond angles (°) | 0.0057  1.16 | 0.0055  1.17 | 0.0059  1.16 | 0.0075  1.23 |
| Validation  MolProbity score  Clashscore  Poor rotamers (%) | 1.47  3.73  0.66 | 1.96  4.69  2.56 | 1.75  5.02  1.45 | 1.91  6.57  1.60 |
| Ramachandran plot  Favored (%)  Allowed (%)  Disallowed (%) | 95.51  4.33  0.16 | 93.78  5.41  0.82 | 94.81  4.38  0.81 | 94.26  5.30  0.44 |

^a^ Range of the per-particle defocus estimates. This range encompasses the defocus values for 90% of the particles used, with 5% of the particle defocuses falling below and 5% exceeding this range.

^b^ Symmetry is imposed during the initial symmetrical refinement preceding the local refinements.

^c^ Total number of particles after symmetry expansion.

^d^ Number of asymmetric units after 3D classification, corresponding to a kinesin motor bound to a tubulin dimer.

**Supplementary Table 1. Cryo-EM data collection, refinement and validation statistics (3/3)**

|  | **MT-KIF1A^P305L^**  **-ANP-TL_012*_**  (EMDB-42550)  (PDB 8UTU) | **MT-KIF1A^P305L^**  **-ADP**  (EMDB-42551)  (PDB 8UTV) | **MT-KIF1A^P305L^**  **-APO**  (EMDB-42552)  (PDB 8UTW) |
| --- | --- | --- | --- |
| **Data collection and processing** |  |  |  |
| Magnification (actual) | 59242 | 57078 | 59242 |
| Voltage (kV) | 300 | 300 | 300 |
| Electron exposure (e^–^/Å^2^) | 49.4 | 62.9 | 50.4 |
| Defocus range (μm) ^a^ | 1.0 ; 2.4 | 0.8 ; 2.0 | 1.2. ; 2.2 |
| Pixel size (Å) | 0.844 | 0.876 | 0.844 |
| Symmetry imposed ^b^  Rise (Å)  Twist (deg) | Helical  5.64  168.09 | Helical  5.64  168.09 | Helical  5.62  168.09 |
| Particle images identified as 15R symmetry (no.) | 125182 | 83861 | 79145 |
| Particle images in helical reconstruction (no.) | 125182 | 83861 | 79145 |
| Single particles (no.) ^c^ | 1877730 | 1257915 | 1187175 |
| Single particles used (no.) ^d^ | 254197 | 119590 | 620149 |
| Overall resolution (Å)  FSC threshold | 3.0  0.143 | 3.0  0.143 | 3.5  0.143 |
| Kinesin resolution (Å) | 3.3 | 3.8^e^ | 4.1 |
| Tubulin resolution (Å) | 3.0 | 2.9 | 3.4 |
|  |  |  |  |
| **Refinement** |  |  |  |
| Model composition  Non-hydrogen atoms  Protein residues  Ligands | 13615  1709  8 | 13338  1679  7 | 9787  1227  4 |
| R.m.s. deviations  Bond lengths (Å)  Bond angles (°) | 0.0064  1.23 | 0.0058  1.18 | 0.0056  1.16 |
| Validation  MolProbity score  Clashscore  Poor rotamers (%) | 1.68  5.23  1.50 | 1.53  4.76  0.70 | 1.97  5.96  1.89 |
| Ramachandran plot  Favored (%)  Allowed (%)  Disallowed (%) | 96.06  3.70  0.24 | 95.87  4.07  0.06 | 93.28  6.47  0.25 |

^a^ Range of the per-particle defocus estimates. This range encompasses the defocus values for 90% of the particles used, with 5% of the particle defocuses falling below and 5% exceeding this range.

^b^ Symmetry is imposed during the initial symmetrical refinement preceding the local refinements.

^c^ Total number of particles after symmetry expansion.

^d^ Number of asymmetric units after 3D classification, corresponding to a kinesin motor bound to a tubulin dimer.

^e^ Note that in this map kinesin density is weak and of low-resolution with no side-chains resolved unlike in the tubulin part of the maps. As a consequence, the kinesin resolution given is an overestimate dominated by the signal near the microtubule interface (see Supplementary Fig. 6).
