# Supplementary material for: Cryo-EM unveils kinesin KIF1A’s processivity mechanism and the impact of its pathogenic variant P305L
Source: Nat Commun. 2024 Jul 2;15:5530. doi: 10.1038/s41467-024-48720-4 (PMC11219953; doi:10.1038/s41467-024-48720-4)
Supplement: Supplementary file 4 — Source Data [file 41467_2024_48720_MOESM4_ESM.zip › Source_Data/Supplementary_Table_2.docx]

| **Dataset** | **Kinesin concentration (µM)** | **Buffer** | **Decorated (%)** | **Undecorated (%)** | **Unknown (%)** |
| --- | --- | --- | --- | --- | --- |
| MT-KIF1A-ANP | 20 | BRB80 | 74 | 21 | 5 |
| MT-KIF1A-ADP | 40 | BRB80 | 32 | 32 | 36 |
| MT-KIF1A-APO | 40 | BRB80 | 95 | 0 | 5 |
| MT-KIF1A^P305L^-ANP | 40 | BRB36 | 25 | 75 | 0 |
| MT-KIF1A^P305L^-ADP | 40 | BRB36 | 9 | 86 | 5 |
| MT-KIF1A^P305L^-APO | 40 | BRB36 | 91 | 9 | 0 |

**Supplementary Table 2. Levels of decoration in each of the cryo-EM datasets.** The decorated fraction corresponds to the proportion of the particles images assigned to class(es) for which the class average(s) after the focused 3D classification on the single kinesin site (or site T for ANP datasets) shows a density that could be recognized as being a kinesin motor domain bound to the tubulin dimer. The undecorated fraction corresponds to class averages showing a lack of kinesin motor domain present on the tubulin dimer. In most datasets, there are some low-resolution classes (like Class 6 in Supplementary Fig. 2) for which the class averages show a density that was not reliably assigned as decorated or undecorated and such cases are listed as unknown in the table. Kinesin concentrations shown in the second column correspond to the concentration of KIF1A monomers (single polypeptide).
