# Supplementary material for: Cryo-EM unveils kinesin KIF1A’s processivity mechanism and the impact of its pathogenic variant P305L
Source: Nat Commun. 2024 Jul 2;15:5530. doi: 10.1038/s41467-024-48720-4 (PMC11219953; doi:10.1038/s41467-024-48720-4)
Supplement: Supplementary file 4 — Source Data [file 41467_2024_48720_MOESM4_ESM.zip › Source_Data/Supplementary_Table_3.docx]

| **Formula and datasets** | ***f* (2HB)**  **(%)** | ***f* (1HB)**  **(%)** | ***f** (2HB)**  **(%)** | ***f** (1HB)**  **(%)** |
| --- | --- | --- | --- | --- |
| Formula | $\frac{100 \times n\left( \text{2HB} \right)}{n\left( \text{1HB}_{\text{all}} \right)+n\left( \text{2HB} \right)}$ | $\frac{100 \times n\left( \text{1HB}_{\text{all}} \right)}{n\left( \text{1HB}_{\text{all}} \right)+n\left( \text{2HB} \right)}$ | $\frac{100 \times n\left( \text{2HB} \right)}{n\left( \text{1HB}_{\text{free}} \right)+n\left( \text{2HB} \right)}$ | $\frac{100 \times n\left( \text{1HB}_{\text{free}} \right)}{n\left( \text{1HB}_{\text{free}} \right)+n\left( \text{2HB} \right)}$ |
| MT-KIF1A-ANP | 36 | 64 | 67 | 33 |
| MT-KIF1A^P305L^-ANP | 58 | 42 | 58 | 42 |
| MT-KIF1A^P364L^-ANP | 38 | 62 | 49 | 51 |

**Supplementary Table 3. Relative frequency of two-heads and one-head bound configurations in the ANP datasets.** The relative abundance of the two-heads-bound configurations (2HB) and of all the one-head-bound configurations (1HB_all_) are provided in the first two columns and named *f*(2HB) and *f*(1HB) respectively. However, because of the molecular crowding on the microtubule, some of the observed one-head-bound-configurations - not all - are due to the fact two consecutive tubulin dimer were not available for binding a two-heads-bound configuration. This is shown in Supplementary Fig 3c where 2 motors in a closed state are bound consecutively (to compare with Supplementary Fig 3b showing bona fide one-heads-bound configurations). The number of two-heads-bound configurations is therefore underestimated with *f*(2HB). It is not possible to know exactly how many more two-heads-bound state would be seen if the decoration of the dataset would be much sparser. However, an estimate can be provided by excluding from the count the one-head-bound state that are clearly in a crowded area (as in Supplementary Fig 3c), keeping only the unambiguous ones (1HB_free_) like in Supplementary Fig 3b. This is what *f**(2HB) and *f**(1HB) provide. Classes with associated class averages at low resolution and/or for which the one-head-bound or two-heads-bound configuration status was unclear were not included in these estimates.
